# Draft Genome Sequences of Four *Aspergillus* Section *Fumigati* Clinical Strains

**DOI:** 10.1128/MRA.00856-20

**Published:** 2020-10-01

**Authors:** Renato Augusto Corrêa dos Santos, Olga Rivero-Menendez, Jacob L. Steenwyk, Matthew E. Mead, Gustavo Henrique Goldman, Ana Alastruey-Izquierdo, Antonis Rokas

**Affiliations:** a Departmento de Ciências Farmacêuticas, Faculdade de Ciências Farmacêuticas de Ribeirão Preto, Universidade de São Paulo, Ribeirão Preto, São Paulo, Brazil; b Department of Biological Sciences, Vanderbilt University, Nashville, Tennessee, USA; c Medical Mycology Reference Laboratory, National Center for Microbiology, Instituto de Salud Carlos III, Madrid, Spain; University of Maryland School of Medicine

## Abstract

*Aspergillus* fungi in section *Fumigati* include important human pathogens. Here, we sequenced the genomes of two strains of Aspergillus hiratsukae and two strains of Aspergillus felis. The average genome sizes are 29.5 Mb for *A. hiratsukae* and 31.8 Mb for *A. felis*.

## ANNOUNCEMENT

*Aspergillus* is a highly diverse genus of industrially and medically important fungi (1, 2). The genus is taxonomically divided into 27 sections (3). Section *Fumigati* contains the major human pathogen Aspergillus fumigatus (4) and several so-called cryptic species, such as Aspergillus hiratsukae and Aspergillus felis (5–7), which are morphologically similar but genetically distinct from A. fumigatus. Cryptic species account for over 10% of cases of *Aspergillus* infection (8). Here, we sequenced the genomes of two clinical strains of *A. hiratsukae*, CNM-CM5793 and CNM-CM6106, from nail and ear infections, respectively, both from Spain. We also sequenced two clinical strains of *A. felis*, strain CNM-CM7691 from an ear infection in Spain and strain CNM-CM5623 from Portugal. All four isolates were recovered from clinical samples following standard procedures and sent to the Medical Mycology Reference Laboratory (at the National Center for Microbiology, Instituto de Salud Carlos III, Madrid, Spain) for identification and susceptibility testing. Except for infection type, no clinical data were recorded. Therefore, the fungal isolates were judged to be exempt from informed consent of the patients and institutional review board approval.

**FIG 1:**
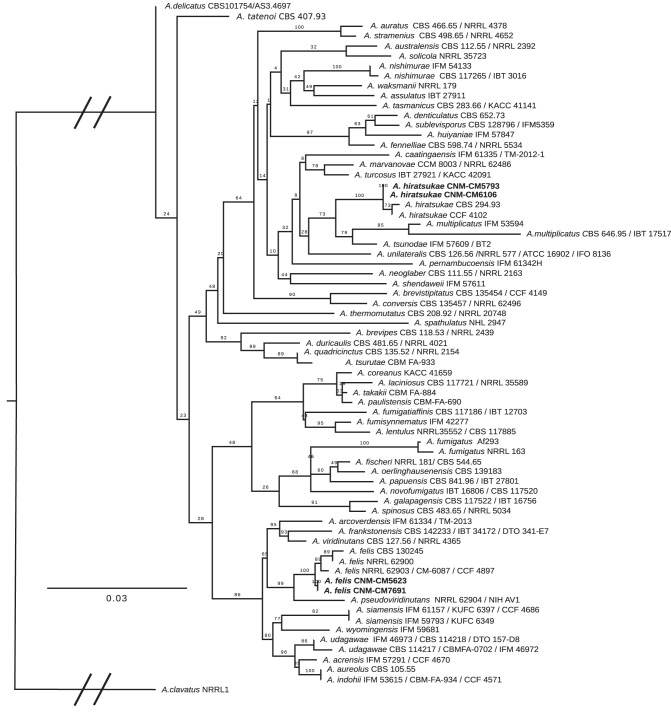
Maximum-likelihood phylogenetic tree of the four strains sequenced in this announcement (in bold) and related species in section *Fumigati*, based on the analysis of the four markers beta-tubulin gene (*benA*), calmodulin gene (*CaM*), actin gene (*act*), and RNA polymerase II second-largest subunit gene (*RPB2*), commonly used in *Aspergillus* taxonomy (18); sequences were obtained from reference 18 except for the sequences of the four newly sequenced strains, which were obtained by searching for markers of each strain in orthogroups generated by OrthoFinder v2.3.3 (19) using A. fumigatus Af293 (17) as the reference. Each marker was aligned with MAFFT v7.397 (20), and a supermatrix was generated with FASconCAT v1.11 (21). Tree inference was carried out on IQ-TREE v2.0.3 (22) with partitions with the option “MFP+MERGE,” which employs ModelFinder to find the best partition scheme. The final tree was edited in FigTree v1.4.4 (http://tree.bio.ed.ac.uk/software/figtree/). Support values are based on 1,000 bootstrap replicates. A. clavatus (section *Clavati*) was used to root the tree. Note that the species A. parafelis, A. pseudofelis, and *A. felis* were merged (synonymized) into a single species, *A. felis* (18); thus, we infer that the two sequenced strains belong to *A. felis*.

Species assignment was based on a maximum-likelihood phylogenetic analysis (Fig. 1). For genome sequencing, we grew all strains in glucose-yeast extract-peptone (GYEP) liquid medium (0.3% yeast extract and 1% peptone; Difco, Soria Melguizo) with 2% glucose (Sigma-Aldrich, Spain) for 24 to 48 h at 30°C. The mycelium was mechanically disrupted by vortex mixing with glass beads and used to extract genomic DNA using the phenol-chloroform method (9). DNA was quantified using the QuantiFluor double-stranded DNA (dsDNA) system and the QuantiFluor ST fluorometer (Promega, Madison, WI, USA). DNA quality was checked with the Agilent 2100 bioanalyzer (Agilent Technologies, Inc., Santa Clara, CA, USA). DNA libraries were prepared using the Nextera DNA library prep kit (Illumina, Inc., San Diego, CA, USA) according to the manufacturer’s guidelines. Paired-end sequencing (2 × 150 bp) was performed using the NextSeq 500 platform following the manufacturer’s protocols (Illumina, Inc.).

For all software, default parameters were used except where otherwise noted. The numbers of sequencing read pairs generated for strains CNM-CM5793, CNM-CM6106, CNM-CM7691, and CNM-CM5623 were 7,733,508, 5,237,901, 9,555,248, and 6,768,577, respectively. Quality control of the sequence reads was performed with FastQC v0.11.7 (https://www.bioinformatics.babraham.ac.uk/projects/fastqc/). Raw reads were cleaned with Trimmomatic v0.38 (10) with the following parameters: NexteraPE-PE.fa:2:30:10:2:keepBothReads, SLIDINGWINDOW:4:15, LEADING:3, TRAILING:3, and MINLEN:90. The genome sequences were assembled with SPAdes v3.14.0 (11) employing multiple k-mers (31, 41, 51, 61, 71, 81, and 91) and the --careful parameter. The genomic reads were mapped to the assembly with Bowtie v2.3.4.1 (12), followed by a single iteration of Pilon v1.22 (13) used in base correction to fix misassemblies and for gap filling. Overall statistics of the final assemblies were assessed with QUAST v4.6.3 (14). Genome assembly completeness was assessed by examining for the presence of 3,546 universal single-copy orthologs in Eurotiomycetes (eurotiomycetes_odb10) with BUSCO v4.0.4 (15). We used AUGUSTUS v3.3.1 (16) for prediction of protein-coding genes using the A. fumigatus Af293 gene models as a reference (17).

Assembly sizes, numbers of contigs, GC content, *N*_50_ contig values, gene numbers, percentages of complete and single-copy BUSCOs, and percentages of fragmented BUSCOs for all four strains are reported in Table 1. We summarized the genome statistics based on contigs of greater than 1,000 bp but included all contigs of greater than 200 bp in the assemblies submitted to GenBank. Genomic information of *Aspergillus* strains that are closely related to major human pathogens is important for understanding the origin and evolution of opportunistic human pathogenicity in the genus *Aspergillus*.

**TABLE 1:** Overall genome assembly, completeness, and annotation statistics

Strain	Assembly size^*a*^ (bp)	No. of contigs >1,000 bp	Avg genome coverage (×)	GC content (%)	*N*_50_ (bp)	No. of genes	No. (%) of complete and single-copy BUSCOs	No. (%) of fragmented BUSCOs
*A. hiratsukae* CNM-CM5793	29,562,918	745	63	50.38	100,935	9,685	3,487 (98.33)	31 (0.87)
*A. hiratsukae* CNM-CM6106	29,374,270	922	39	50.37	71,695	9,663	3,466 (97.74)	38 (1.07)
*A. felis* CNM-CM5623	31,643,783	663	47	49.93	112,776	10,161	3,494 (98.53)	26 (0.73)
*A. felis* CNM-CM7691	31,957,614	559	70	49.93	138,232	10,243	3,503 (98.78)	18 (0.51)

a Based on contigs with more than 1,000 bp.

### Data availability.

The draft genome sequences of *A. felis* strains CNM-CM5623 and CNM-CM7691 and *A. hiratsukae* strains CNM-CM5793 and CNM-CM6106 are deposited in GenBank under the accession numbers JACBAE000000000, JACBAG000000000, JACBAD000000000, and JACBAF000000000, respectively. The raw reads of *A. felis* strains CNM-CM5623 and CNM-CM7691 and *A. hiratsukae* strains CNM-CM5793 and CNM-CM6106 are deposited in the NCBI Sequence Read Archive (SRA) under accession numbers SRR11804853, SRR11804830, SRR11802685, and SRR11802449, respectively. Genome assemblies and raw data are associated with BioProject number PRJNA633131.
